# GMP Synthetase: Allostery, Structure, and Function

**DOI:** 10.3390/biom13091379

**Published:** 2023-09-12

**Authors:** Lionel Ballut, Sébastien Violot, Sanjeev Kumar, Nushin Aghajari, Hemalatha Balaram

**Affiliations:** 1 Molecular Microbiology and Structural Biochemistry, CNRS, University of Lyon1, UMR5086, 7 Passage du Vercors, CEDEX 07, F-69367 Lyon, France; lionel.ballut@ibcp.fr (L.B.); sebastien.violot@ibcp.fr (S.V.); 2 Trivedi School of Biosciences, Ashoka University, Rajiv Gandhi Education City, Sonipat 131029, Haryana, India; sanjeev.kumar@ashoka.edu.in; 3 Molecular Biology and Genetics Unit, Jawaharlal Nehru Centre for Advanced Scientific Research, Jakkur 560064, Bangalore, India

**Keywords:** GMP synthetase, glutamine amidotransferase, ATP pyrophosphatase, glutaminase, crystal structure, allostery, ammonia channeling, drug target, succinimide

## Abstract

Glutamine amidotransferases (GATs) catalyze the hydrolysis of glutamine and transfer the generated ammonia to diverse metabolites. The two catalytic activities, glutaminolysis and the subsequent amination of the acceptor substrate, happen in two distinct catalytic pockets connected by a channel that facilitates the movement of ammonia. The *de novo* pathway for the synthesis of guanosine monophosphate (GMP) from xanthosine monophosphate (XMP) is enabled by the GAT GMP synthetase (GMPS). In most available crystal structures of GATs, the ammonia channel is evident in their native state or upon ligand binding, providing molecular details of the conduit. In addition, conformational changes that enable the coordination of the two catalytic chemistries are also informed by the available structures. In contrast, despite the first structure of a GMPS being published in 1996, the understanding of catalysis in the acceptor domain and inter-domain crosstalk became possible only after the structure of a glutamine-bound mutant of *Plasmodium falciparum* GMPS was determined. In this review, we present the current status of our understanding of the molecular basis of catalysis in GMPS, becoming the first comprehensive assessment of the biochemical function of this intriguing enzyme.

## 1. Introduction

Guanosine 5′-monophosphate (GMP) synthetase (GMPS, E.C. 6.3.5.2) catalyzes the final step in the *de novo* biosynthesis of GMP and belongs to the glutamine amidotransferase (GAT) family of enzymes. GMP serves as the precursor to GTP that supports key cellular processes such as DNA replication, transcription, and translation, along with being an energy source in many cellular processes. Intracellularly, there are three possible routes for the generation of GMP; guanine phosphoribosylation, catalyzed by hypoxanthine guanine phosphoribosyltransferase (HGPRT); guanosine phosphorylation, catalyzed by guanosine kinase; and xanthosine 5′monophosphate (XMP) amination by GMPS. Guanosine kinase is largely restricted to certain bacteria, whereas HGPRT and GMPS have been found to be present in many organisms, including archaea, prokaryotes, and eukaryotes. Despite the presence of alternate cellular pathways for GMP synthesis, GMPS is an essential enzyme in many pathogenic organisms, making the enzyme a key drug target.

## 2. GMPS: An Essential Metabolic Enzyme

The essentiality of GMPS in *Plasmodium berghei* and *P. falciparum* has been established through reverse genetics [1,2], and bredinin, an inhibitor of GMPS and inosine monophosphate dehydrogenase, the preceding enzyme in the GMP biosynthesis pathway, exhibits potent antimalarial activity [3,4].

This is also seen in *Trypanosoma brucei*, where *gmps* null parasites have failed to establish infection in mice and validate the enzyme as a potential therapeutic target for human African trypanosomiasis [5]. In *Candida albicans* and *Aspergillus fumigatus*, both medically significant fungal pathogens, GMP synthase activity is required for virulence. The rescue by exogenous guanine of the effect of the small molecule ECC1385 inhibitor of fungal GMPS activity on *C. albicans* led to the identification of the enzyme as an anti-fungal drug target [6]. Similarly, GMP synthetase has been shown to be necessary for virulence factor production and infection by *Cryptococcus neoformans*, an opportunistic pathogen that establishes infection in immunocompromised AIDS patients [7]. The deletion of the operon that codes for IMPDH and GMPS in *Shigella flexneri* yielded an attenuated auxotrophic strain that conferred immunogenicity in volunteers, thus serving as a vaccine candidate for Shigellosis, a diarrheal disease [8,9].

For the *in vitro* growth of *Mycobacterium tuberculosis* H37Rv, GMPS has been shown to be essential [10]. In *Clostridioides difficile*, the bacterium that causes life-threatening diarrhea, the expression of GuaA, which codes for GMPS, is controlled by a riboswitch that is responsive to levels of guanine. An impairment in the growth of *C. difficile* and its reduced capacity to colonize the mouse gut upon the inactivation of GMP synthetase demonstrates the importance of *de novo* GMP biosynthesis and the possibility of targeting guanine riboswitches with suitable analogues [11]. In rapidly proliferating cells, there is increased activity of the enzymes involved in nucleotide metabolism due to the need for increased levels of nucleotides, making these enzymes, including GMPS, potential drug targets for both anti-cancer and immunosuppressive chemotherapies [12,13,14,15].

### Moonlighting Functions of GMPS

Apart from catalyzing GMP synthesis, GMPS has been found to perform several other non-catalytic moonlighting functions. Under the conditions of xenotoxic stress or upon nucleotide deprivation, GMP synthetase is translocated to the nucleus, where it mediates p53 stabilization [16]. GMPS has been found to bind ubiquitin-like domains of HUBL-123 and hyperactivate ubiquitin-specific protease 7 (USP7) *via* the stabilization of its HUBL-45-dependent active state [17]. The role of the GMPS-USP7 complex has been demonstrated in the transcriptional regulation of the viral EBNA-1 protein during the latent phase of infection with Epstein–Barr virus [18]. Studies on Drosophila GMPS have shown that the enzyme interacts with USP7 (ubiquitin-specific protease 7) and deubiquitylates histone H2B, which, in turn, has important implications in gene regulation [19]. The USP7-GMPS complex has been reported to bind to gene loci that are regulated by the steroid hormone ecdysone and has been proposed to act as a co-repressor of the developmental genes controlled by ecdysone [20]. In addition, it has been observed that Usp7 forms a complex with GMPS to promote Hedgehog pathway activity [21]. Moreover, the GMP synthetase gene fused to the MLL (mixed-lineage leukemia) gene was detected in a patient with metastatic neuroblastoma, but the functional implications of this fusion on the GMPS and MLL functions are still not well understood [22]. 

## 3. Biochemical and Kinetic Characteristics of GMPSs

GMPS belongs to the glutamine amidotransferase (GAT) family of enzymes that enable the biosynthesis of nitrogen-containing molecules through the transfer of ammonia derived from the amide of glutamine to various acceptor substrates [23,24,25,26]. 

GATs are involved in the biosynthesis of amino acids, amino sugars, purine and pyrimidine nucleotides, co-enzymes, and antibiotics [26] and play a central role in metabolism. The enzymes of this family are modular in organization, with each module corresponding to a distinct domain or subunit. The glutaminase or amidotranferase (GATase) module harbors the active site where glutamine hydrolysis occurs, leading to the generation of ammonia that is channeled to the active site in the synthetase domain, resulting in the amination of the acceptor substrate. The two active sites are spatially separated by 10–40 Å [27]. Apart from the ammonia generated from glutamine, most GATs can also use exogenous ammonia [24,25,26]. The glutamine hydrolysis in most GATs is tightly regulated by cues from the acceptor/synthetase domain. Ligand binding in the acceptor domain induces conformational changes that have key implications in GATase activation and ammonia channeling [28,29,30,31,32,33].

**Figure 1:**
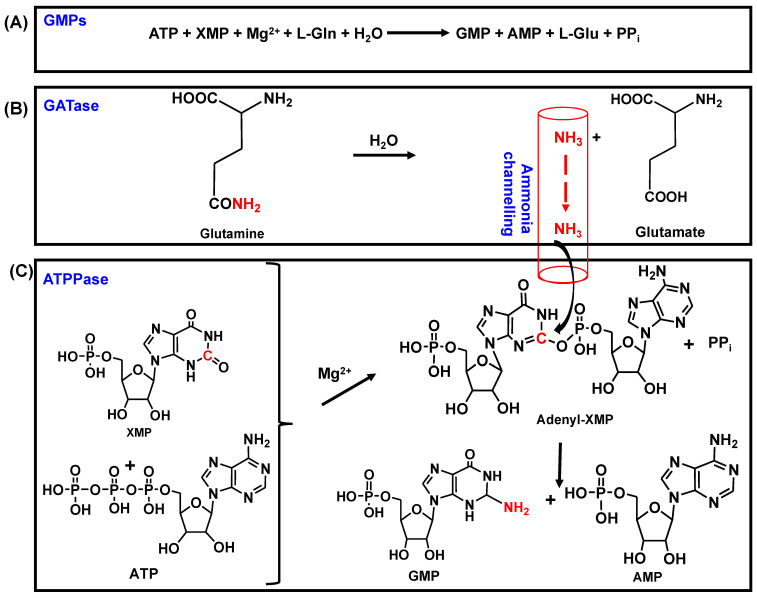
The reaction catalyzed by GMP synthetases. (**A**) Overall reaction, (**B**) reaction in the GATase domain, and (**C**) reaction in the ATPPase domain.

GMPS is a G-type or class I triad amidotransferase that catalyzes the amination of XMP to yield GMP, in a reaction that utilizes glutamine and ATP and through an adenyl-XMP (AMP-XMP) intermediate [26,34,35] (see Figure 1). 

This reaction comprises two distinct catalytic chemistries that operate at two different sites on the enzyme, *viz*., the glutaminase or glutamine amidotransferase (GATase) site for glutamine hydrolysis and the ATP pyrophosphatase (ATPPase) site for the formation of GMP. In GMP synthetases from bacteria and eukaryotes, the GATase and ATPPase units are two domains of a polypeptide chain (two-domain type), whereas, in many archaea, the two units are independent proteins (two-subunit type) coded for by two different genes [36,37]. 

The ATPPase module binds a molecule of ATP.Mg^2+^ and XMP and catalyzes the formation of the adenyl-XMP intermediate [25,26]. *In vitro*, the two-domain-type GMP synthetases, as well as the ATPPase subunit of the two-subunit-type enzymes, can also utilize external ammonia to synthesize GMP [24]. In the GMPSs studied thus far, the GATase domain/subunit is inactive or weakly active, and the binding of ATP.Mg^2+^ and XMP to the ATPPase domain/subunit allosterically activates the GATase domain/ subunit, leading to glutamine (Gln) binding and hydrolysis. The ammonia thus generated is channeled to the ATPPase active site, where, through a nucleophilic attack of adenyl-XMP, GMP is generated [7,31,32,34,36,37,38,39,40].

**Figure 2:**
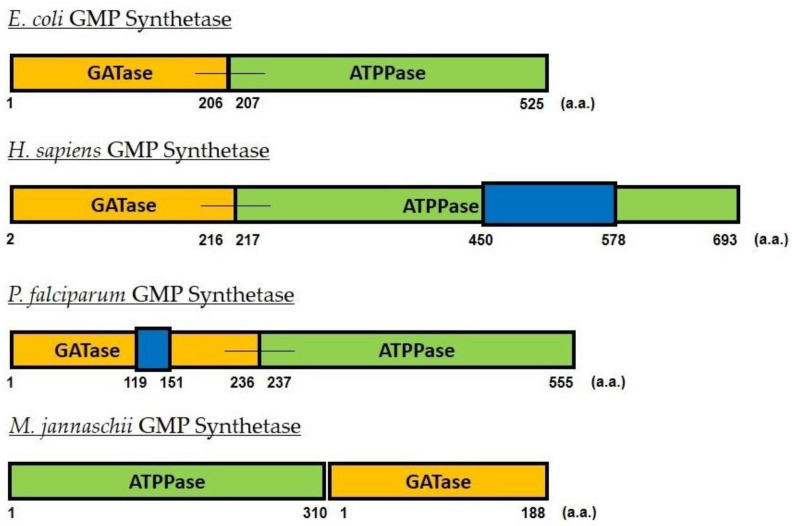
Schematic of the domains and subunits in the gene and protein sequences of GMPS from *E. coli*, *H. sapiens*, *P. falciparum*, and *M. jannaschii.* The bacterial, human, and parasite GMPSs are single-gene proteins, which encode the GATase domain (orange) at the N-terminus and the ATPPase domain (green) at the C-terminus. Insertions in the GATase domain of the *P. falciparum* and in the ATPPase domain of *H. sapiens* enzymes are represented in blue. The *Mj*GMPS is a two-gene protein, encoding the ATPPase subunit (green) and a GATase subunit (orange). In the chromosome of *M. jannaschii*, the gene for the ATPPase subunit precedes that of the GATase subunit, as reflected in the figure.

*Escherichia coli* (*Ec*) GMPS, which is typical of prokaryotic GMPS, is 525 amino acids long with 1 to 201 comprising the GATase domain and linked *via* a short linker to the ATPPase domain (208 to 525 amino acid residues), with the latter containing a dimerization sub-domain [41].The mammalian GMPSs, including that from humans, have an insertion of about 100 amino acids in the so-called dimerization domain [42], whereas fungal and protozoal GMPSs lack this insertion and are similar to prokaryotic GMPS [32,43]. Many thermophilic archaea, including *Pyrococcus horikoshii* (*Ph*) and *Methanocaldococcus jannaschii* (*Mj*), have the two domains expressed as two different subunits. These are similar to the two domains of *E. coli* GMPS, in that they lack the insertion in the dimerization domain [36,37]. Human GMPS is a monomer in solution, while those from *E. coli*, *P. falciparum* (*Pf*), *Mycobacterium tuberculosis* (*Mtb*), and *Aspergillus fumigatus* (*Af*) are dimers [34,38,40,43,44]. *Ph* and *Mj* ATPPases are dimers and associate with two monomeric units of GATase [36,37] (Figure 2). A sequence alignment of representatives of these types of GMPSs is given in Supplementary Figure S1.

Table 1 provides the kinetic parameters of all the GMPSs that have been studied thus far. These GMPSs utilize the ammonia generated from the hydrolysis of glutamine and also exogenous ammonia for the amination of XMP. In all cases, the v *vs*. [ATP] plots were hyperbolic, with the *K*_m_ values ranging from 27 to 452 μM and *Mtb* and *Mj*GMPS exhibiting the lowest and highest values, respectively. XMP exhibited sigmoidal kinetics, with Hill coefficient values of 1.48 and 2.4 for human and *Mtb* GMPS, respectively. The remaining GMPSs that have been studied do not show cooperative behavior in the binding of XMP. The *K*_m_ (*K*_0.5_) values for XMP range from 8.8 to 166 μM and are largely below that of ATP. Like ATP, Gln also exhibits hyperbolic kinetics, with *K*_m_ values ranging from 240 μM to 2.69 mM across the enzymes studied. The *K*_m_ value for NH_4_Cl shows a large variation, with the highest value of 174 mM observed for human GMPS. Except for *Mtb* GMPS, which shows a slight sigmoidicity for NH_4_^+^, the rest follow non-cooperative kinetics. The *k*_cat_ values show variation from 23 to 0.43 s^−1^, with *Pf*GMPS exhibiting the lowest turnover. The significantly lower *K*_m_ value for Gln over NH_4_^+^ in all the GMPSs studied shows that Gln is the preferred physiological substrate.

All the GMPSs studied thus far require Mg^2+^ for activity, and, except for *Af*GMPS, the rest show a positive homotropic cooperativity for the metal ion, with Hill coefficient values ranging from 2.05 to 4.4 and indicating an additional binding site for Mg^2+^, apart from ATP.Mg^2+^ (Table 1 and references therein). Studies on the *Mj*ATPPase subunit of the two-subunit-type GMPS have shown that the synthetase subunit alone has an additional binding site for Mg^2+^, with NMR studies on *Mj*GATase indicating weak interactions with Mg^2+^ being associated with this subunit too [37,45].

*Pf*GMPS exhibits a steady-state ordered binding of ATP followed by XMP to the ATPPase domain, with glutamine binding in a random manner to the GATase domain. The irreversible Ping Pong step seen in initial velocity kinetics has been attributed to the release of glutamate before the ammonia attack of the adenyl-XMP intermediate. Product inhibition patterns show the order of release of products, where glutamate is the first product to be released, followed by AMP and GMP, before the final release of PP*_i_* [38]. *Ec*GMPS also follows the ordered addition of MgATP, followed by XMP, with ammonia (glutamine) addition being ordered or partially random, wherein the binding can occur before or after MgATP/XMP [46]. In the case of human GMPS, the inhibition patterns obtained with decoyinine, an analogue of adenosine, with respect to ATP, XMP, and Gln, suggest that ATP may not be the first substrate to bind to the enzyme [34]. Substrate binding monitored by isothermal titration calorimetry has indicated that *Mtb*GMPS binds substrates randomly, with PP*_i_* being the last product released [40]. 

The GATase active site in GMPS has a conserved catalytic triad consisting of a Cys, His, and Glu residue [39,41]. In all the GMPSs studied, both single- and two-subunit types, the activity of the ATPPase module is independent of GATase, whereas the latter cannot catalyze the hydrolysis of glutamine in the absence of substrates [34,38,40,46], with the exceptions of *Pf* and *Ph*GMPS, which exhibit GATase activity even without a complete occupancy of the ATPPase pocket, albeit at low levels [32,36,38,39]. Among the GATs, asparagine synthetase has been shown to have leaky glutaminase activity [47,48]. The allosteric activation of the GATase domain in *Ec*, human, and *Pf*GMPS is also supported by increased rates of inactivation by the irreversible inhibitors, acivicin, and DON upon the full occupancy of the ATPPase active site by the substrates [38,49,50,51]. Tethering the two subunits of *Mj*GMPS does not activate the fused GATase subunit, and the hydrolysis of Gln is observed only in the presence of ATP.Mg^2+^ and XMP [37]. In the case of *Pf* and *Mj*GMPS, ligand binding to the ATPPase domain/subunit with XMP and p[NH]ppA, the latter being a non-hydrolysable analogue of ATP, also leads to an increase in glutaminase activity, while these ligands have no effect on the activity of the GAT domain of human GMPS [34,37,38]. In addition, in *Mj*GMPS, the combination of XMP + pyrophosphate (PP*_i_*) also activates *Mj*GATase, albeit to lower levels [37]. These results highlight the differences in GMPSs, where, in some cases (but not all), AMP-XMP formation is essential for activating the GATase domain/subunit. Interestingly, the phosphorylated-UTP intermediate has a similar activating role in CTP synthase, another glutamine amidotransferase [52].

GMP synthetase converts XMP into GMP with stoichiometric hydrolysis of ATP to AMP and inorganic pyrophosphate, and l-glutamine to l-glutamic acid, suggesting ammonia channeling. pH-dependent studies of glutamine- and ammonia-dependent activities, in conjunction with ^15^N-edited proton NMR spectroscopy carried out on *Pf*GMPS, have established that the ammonia released from glutamine is not equilibrated with the external medium, but is channeled to the ATPPase active site [39]. It has been shown that Gln-dependent GMP formation is at its maximum when the ratio of *Mj*GATase and *Mj*ATPPase is 1:1, and this supports ammonia channeling in two-subunit GMPS [45].

The formation of the AMP-XMP intermediate during the course of the reaction was proposed by positional isotope exchange and kinetic experiments with *Ec*GMPS, wherein the exchange of ^18^O from the non-bridge to bridge position of ATP has been observed only in the presence of XMP and Mg^2+^ [46].

## 4. Crystal Structures of Single- and Two-Chain GMPS 

The available crystal structures of free and ligand-bound GMPSs, GATase domains, and subunits, and ligand-complexed subunits of ATPPase from different organisms are summarized in Table 2. 

**Figure 3:**
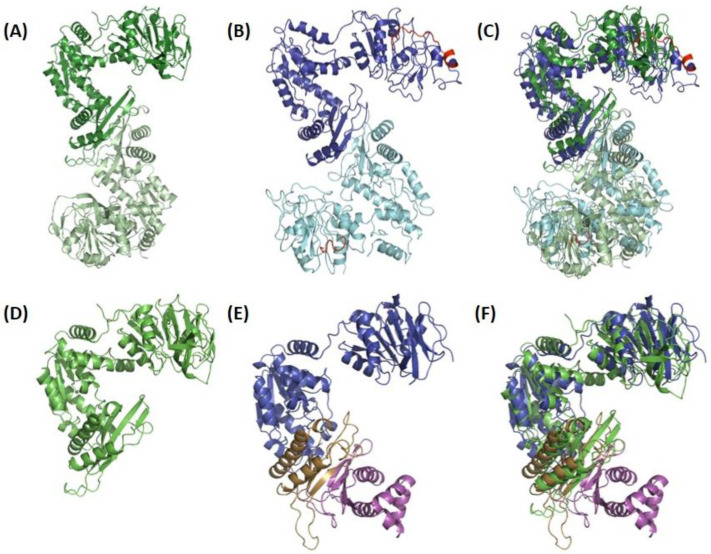
Three-dimensional structure of (**A**) dimeric *Ec*GMPS, with the monomers colored in dark and light green, respectively, (**B**) dimeric *Pf*GMPS, with the monomers colored in dark and light blue, respectively, and the extra “insertion-domain” colored in red, (**C**) overlay of these two dimers, (**D**) monomer A of the dimeric *Ec*GMPS, (**E**) monomeric *Hs*GMPS with the so-called D1 (extra domain) and D2 domains colored in pink and yellow, respectively, and (**F**) overlay of these two monomers. It can be noticed that the D1 and D2 sub-domains mimic the dimer interface, as observed in dimeric GMP synthetases.

All single-chain GMPS structures have two catalytic domains, the GATase and ATPPase domains, connected by a short linker that is 12 residues long in *Pf*GMPS. The synthetase domain is further subdivided into two sub-domains, the N-terminal catalytic core and C-terminal dimerization sub-domains, a feature seen in *Ec*GMPS and in all the other available GMPS structures, except that of human (Figure 3, PDB-IDs are given in Table 2) [33,40,41,43,46]. This latter enzyme has a 100 amino acid residue insertion in the ATPPase domain, resulting in the N-terminal sub-domain, followed by two dimerization sub-domains. Of the available single-chain, full-length GMPS structures, five are without ligands and seven are with bound ligands, and of these, only the *Pf*GMPS structure has a ligand bound in its GATase domain. Concerning two-subunit GMPS structures, two are without ligands and two are with bound ligands. Details are given in Table 2. The available structures of all GMPSs that are like *Ec*GMPS in their sequence, native or ligand-bound are similar. However, the structures of Gln-bound *Pf*GMPS_C89A and unliganded *Pf*GMPS_C89A/C113A are exceptions due to the different tertiary and quaternary structure organizations of the two domains as compared to the native structure. All of these GMPSs, like *Ec*GMPS, are dimers in solution, and the biological assembly in the crystal structure is also a dimer (Figure 3) [32,38,41,43,53]. 

In the crystal, *Pf*GMPSC89A/C113A and *Pf*GMPSC89A/Gln only display one molecule in the asymmetric unit, as opposed to in other forms. It should be mentioned that, while the dimer of *Pf*GMPSC89A/Gln can be generated *via* symmetry, this is not possible for *Pf*GMPSC89A/C113A [32,33]. Although the crystal structure of human GMPS shows association into dimers, the enzyme is a monomer in solution under both liganded and non-liganded conditions [34,42,54]. 

The structures of the ATPPase and GATase subunits of *Mj* and *Ph* are similar to the corresponding domains of the single-chain GMPS [36,37]. *Mj* and *Ph*GATase are monomers in solution and the biological assembly in the crystal is a monomer in *Ph*GATase and a dimer in *Mj*GATase [36,45,55,56]. 

The ATPPases of *Mj* and *Ph* are dimers in both the crystal and solution, and the subunit association through the dimerization interface is similar to that observed in dimeric GMPS [36,37]. 

### 4.1. Structure of the GATase Domain/Subunit and Catalysis of Gln Hydrolysis

The GATase domain in GMPS is an independent structural domain with an α/β fold, consisting of nine to twelve β-strands surrounded by five α-helices. In *Pf*GMPS, the GATase domain has a unique insertion of 32 amino acid residues inserted between β5 and β6 [32]. The isolated *Pf*GATase is monomeric in solution and the structures of the isolated domains of the *Pf* and human (4WIN and 2VPI) GMPSs are similar to those observed in the full-length enzymes [32,42]. *Pf*GMPS and the *Pf* and *Ph*GATase domains/subunits show weak glutaminase activity [32,36,38], and a comparison of the *K*_m_ values of Gln for the unfused *Pf*GATase and unliganded *Pf*GMPS with that for the substrate-bound enzyme shows more than a 280-fold increase in affinity for glutamine upon ligand binding, indicating K-type (change in *K*_m_) activation [32]. The catalytic residues Cys89, His208, and Glu210, which form a catalytic triad in the *Pf*GMPS GATase active site, are invariant across GMPS sequences and similar to the triad in many hydrolytic enzymes [57,58]. 

**Figure 4:**
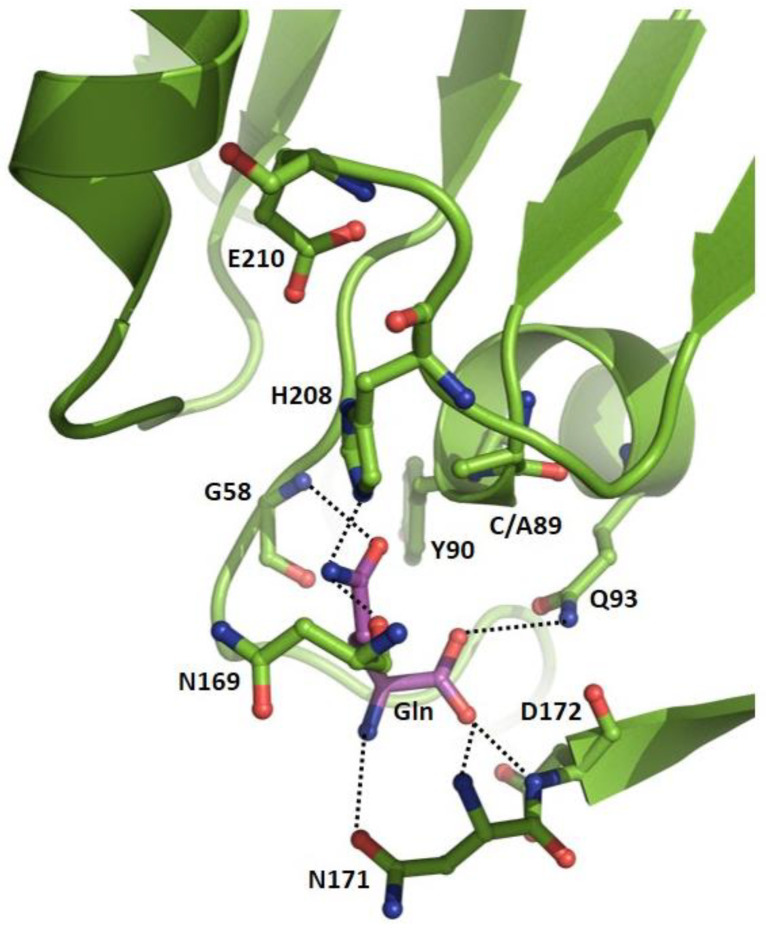
Close-up of the GATase active site of the *Pf*GMPS_C89A/Gln complex (PDB-ID 4WIO).

The only crystal structure that informs on the interactions of Gln with the active site residues is that of the *Pf*GMPS_C89A/Gln complex [32] (Figure 4).

The hydrogen bonding interactions between the carboxyl oxygens of the substrate, the side-chain amide NH_2_ of Gln93, and the main-chain amide NH of Asn171 and Asp172 orient the substrate glutamine in the catalytic pocket. The substrate amide carboxyl oxygen is in close proximity to the main-chain NHs of Gly58 and Tyr90, while the substrate amide NH_2_ hydrogen bonds with the main-chain carbonyl of Asn169 and the side-chain of catalytic His208, the general base that abstracts a proton from Cys89. In addition, the substrate Gln α amino NH_2_ contacts the side-chain amide CO of Asn171. Together, these interactions serve to orient the substrate glutamine within hydrogen-bonding distance (3.3 Å) of the catalytic Cys89, with the whole assembly mimicking the pre-catalytic state of the GATase domain [32] (Figure 4).

In all triad GATs, a conserved Gln and highly invariant Asp/Glu residue, which interact with the α carboxyl O and α amino N, respectively, of the substrate Gln, hold the molecule in the right orientation for the hydrolysis of the side-chain amide [24,25,27]. In *Pf*GMPS, this interaction, mediated by Gln93, is evident in the structure of the *Pf*GMPS_C89A/Gln complex [32]. The importance of this interaction has been established by studies on the mutant of the corresponding residue in *Ec*CPS, also a class I triad GAT [59]. Asp172, which is conserved as Asp or Glu in all GMPSs and lines the Gln-binding pocket, has its side-chain carboxylate oxygen atoms distal (>5 Å) from the α amino nitrogen of the substrate Gln in the structure of *Pf*GMPS_C89A/Gln. Mutation of Asp172 to Ala leads to a 170-fold increase in the *K*_m_ value for Gln, supporting its key role in substrate binding. Upon activation enabled by the binding of the substrates to the ATPPase active site, D172 must be appropriately oriented to interact with substrate Gln α amino NH_2_, resulting in an increased binding affinity [32].

### 4.2. Structure of the ATPPase Domain/Subunit

**Figure 5:**
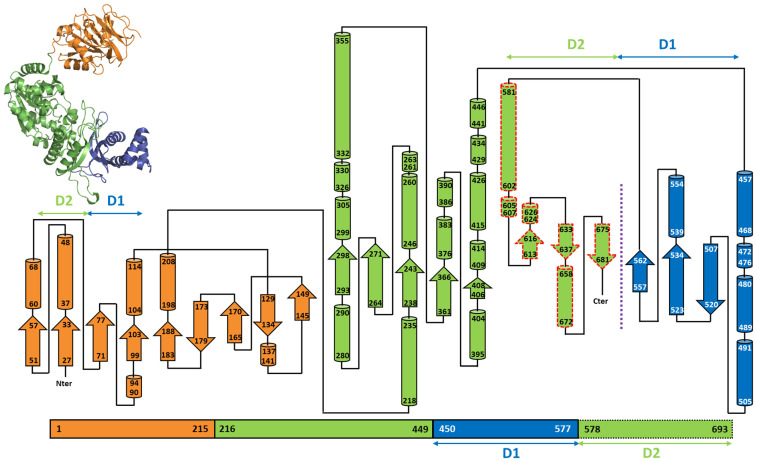
Topology diagram for *Hs*GMPS. The GATase domain is colored in orange, and the ATPPase domain which is divided into three sub-domains: the N-terminal ATP pyrophosphatase domain of 180–200 residues and the C-terminal XMP-binding and dimerization domain of about 120 residues, both in green color. In the ATPPase domain of human GMPS, the presence of an insertion of 127 amino acids (known as the D1 sub-domain) leads to the formation of two dimerization subdomains, with the enzyme being a monomer in solution. The D1 sub-domain (in blue) of *Hs*GMPS, which is lacking in the bacterial, protozoal, and archaeal counterparts, is composed of a 3-stranded antiparallel β-sheet flanked by 5 α-helices.

In single-chain GMPSs, a linker of 10–12 residues connects the GATase to the ATPPase domain, in which the intermediate adenyl-XMP is formed and converted into GMP upon a nucleophilic attack by NH_3_. Isolated domains of *Pf*ATPPase and *Ec*ATPPase retain their activity and utilize externally provided NH_3_ to convert XMP into GMP in the presence of ATP [32,44]. Size-exclusion chromatography has shown that, in solution, both *Pf* and *Ec*ATPPase are dimeric, confirming that dimerization is mediated *via* this domain, a feature that is also observed in the *Mj* and *Ph*ATPPase subunits and in the crystal structures of the respective GMPSs [32,36,37,44]. The ATPPase domain has two sub-domains, the N-terminal ATP-binding domain of 180–200 residues and the C-terminal XMP-binding and dimerization domain of about 120 residues. In the ATPPase domain of human GMPS, the presence of an insertion of 128 amino acids (known as the D1 sub-domain) leads to the formation of two dimerization sub-domains, with the enzyme being a monomer in solution [42] (Figure 5).

**Figure 6:**
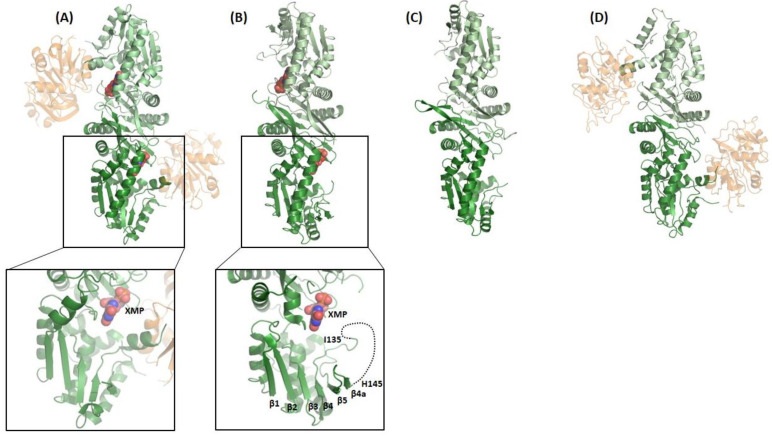
The crystal structures of (**B**) *Mj*ATPPase/XMP and (**C**) *Ph*ATPPase subunits are similar to the structures of the ATPPase domains of two-domain-type *Pf*GMPS (**A**) XMP-bound and (**D**) nonliganded, with the former showing ordering of a significant stretch of the lid loop (see insert in (**A**)). The lid loop in *Mj*ATPPase, spanning amino acids 128−151, that connects the strands β4 and β5 (insert in (**B**)), is ordered, barring residues 136−144, for which the electron density is missing. In this structure, the C-terminal end of the lid loop, comprising residues His145-Leu149, forms a β-strand (β4a) that is antiparallel and hydrogen-bonded to strand β5 of the core β-sheet formed by strands β1–β5. ATPPase domains/subunits are colored in green and GATase domains are colored in beige. XMP is shown as spheres. It should be noticed that, in the close-up views, the helices corresponding to residues 332−356 in (**A**) and residues 86−122 in (**B**) have not been shown in order not to hide information that has been highlighted herein.

**Figure 7:**
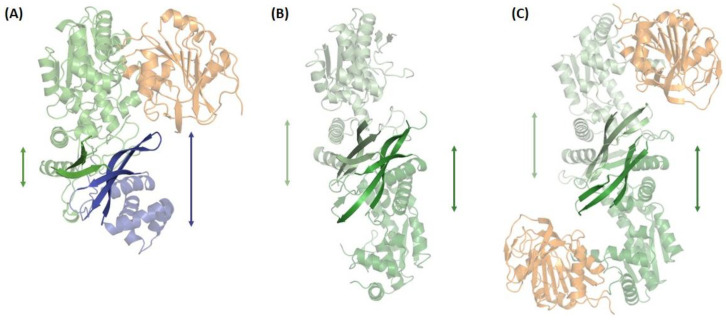
The orientation of D1 (blue) and D2 (green) sub-domains in (**A**) *Hs*GMPS is very similar to the dimer interface formed by the C-terminal sub-domain in (**B**) archaeal ATPPase and (**C**) prokaryotic GMPS (here exemplified by *Mj*ATPPase and *Ec*GMPS, respectively). Domains colored in orange correspond to GATase domains. ATPPase domains from different monomers have been colored in light and dark green, respectively.

The N-terminal sub-domain of ATPPase (Figure 5) has a topology similar to the dinucleotide-binding fold of dehydrogenases and is composed of a twisted, five-stranded, parallel β-sheet sandwiched between helical layers of 9–11 α-helices. The sub-domain has a nucleotide-binding P-loop motif that binds PP*_i_* in the *Ec*GMPS.AMP.PP*i* complex [41]. With the exception of human GMPS, the lid loop following the α-helix, bearing the catalytic residues of the ATPPase domain (residues 376–401 in *Pf*GMPS), is not mapped or partially mapped in most available structures of GMPSs. Although fully mapped in human GMPS, the lid loop being present above and distal from the active site of the ATPPase domain does not throw any light on its possible role in catalysis. With the structure of *Pf*GMPS_C89A/Gln, in which the lid loop that is largely mapped except for a short stretch of eight residues, has moved into the active site in the ATPPase domain; the implications of this conformational dynamics have become clear and are discussed in the following sections. The structures of the *Mj*ATPPase/XMP and *Ph*ATPPase subunits are similar to the structures of the ATPPase domains of two-domain-type native and XMP-bound GMPSs (Figure 6A–D), with the former showing the ordering of a significant stretch of the lid loop [36,37]. The lid loop in *Mj*ATPPase, spanning amino acids 128–151, which connects the strands β4 and β5 (Figure 6E,F and Figure 7), is ordered, barring residues 136−144, for which the electron density is missing. In this structure, the C-terminal end of the lid loop, comprising residues His145-Leu149, forms a β-strand (β4a) that is antiparallel and hydrogen-bonded to strand β5 of the core β-sheet formed by strands β1 to β5 (Figure 6) [37]. 

The ATPPase C-terminal sub-domain of 108–115 residues is involved in dimerization [32,36,37,41,43] and is connected to the ATPPase N-terminal core sub-domain by a linker that, in *Mj*ATPPase, is 17 residues long [37]. This sub-domain in *Pf*GMPS is composed of a three-stranded, parallel β-sheet from each subunit, forming a barrel surrounded by helices (Figure 6). The residues involved intermolecular interactions are largely conserved in dimeric GMPS, but not in human GMPS, which has a 100 amino acid insertion, referred to as the D1 domain, between the N-terminal synthetase and C-terminal dimerization domains. This D1 sub-domain of human GMPS that is absent in the bacterial, protozoal, and archaeal counterparts is composed of a three-stranded, antiparallel β-sheet flanked by five α-helices (Figure 5). The sub-domain of 115 residues following D1 in human GMPS is referred to as the D2 sub-domain, with the fold of the D1 and D2 subdomains being very similar with an rmsd of 2.2 Å (Supplementary Figure S2). The orientation of the D1 and D2 sub-domains in human GMPS is very similar to the dimer formed by the C-terminal sub-domain in prokaryotic and archaeal GMPSs (Figure 7) [42]. In dimeric GMPS and archaeal ATPPase, the dimeric interface participates in XMP binding and the residues involved in this interaction are conserved in the insertion (D1 sub-domain) in monomeric human GMPS (discussed in detail in the following section). The structures of the dimerization sub-domain and the inter-subunit contacts in the dimer of the *Mj* and *Ph*ATPPase subunit structures are similar to the counterparts in two-domain GMPS structures (Supplementary Figure S3) [36,37]. Interestingly, in the *Mj*ATPPase/XMP structure, the two monomers are cross-linked by a disulfide bond involving Cys239, a residue in the C-terminal dimerization sub-domain that is not conserved, barring a few sequences from the genus *Methanocaldococcu*s [37]. In addition, *Ph*ATPPase shows differences of 8° and 5°, respectively, in the relative orientations of the N-terminal and C-terminal sub-domains when compared to *Ec*GMPS and *Tt*GMPS, suggesting the presence of a hinge motion between the sub-domains that may have implications in catalysis [36]. 

### 4.3. The GATase and ATPPase Domains Are Flexible Relative to Each Other

**Figure 8:**
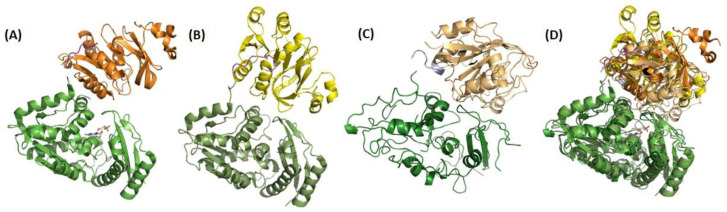
Three-dimensional structures of *Pf*GMPS showing different relative orientations of the GATase and ATPPase domain. In all figures, the ATPPase domains (bottom, in green) are shown in the same relative orientation (**A**) *Pf*GMPS in non-liganded state (PDB-ID 4WIM) and in complex with XMP (3UOW) are shown in the same orientation (here only the XMP (in sticks)-bound structure has been displayed), (**B**) *Pf*GMPS_C89A/Gln (4WIO). The GATase domain is rotated by 85° and translated by 3 Å relative to the ATPPase domain in comparison to 4WIM, (**C**) double-mutant *Pf*GMPS_C89A_C113A (7ZU9). The GATase domain is rotated by 170° and translated by 1.7 Å relative to the ATPPase domain in comparison to 4WIM. (**D**) Superposition of the crystal structures of the single and double mutants onto that of the XMP-bound structure.

The available native AMP.PP_i_- and XMP-bound structures of single-chain GMPSs have a similar orientation of their GATase and ATPPase domains, with an open, solvent-exposed cavity between the two domains. A different orientation of the two domains is observed in *Pf*GMPS_C89A/Gln (PDB-ID 4WIO), wherein the GATase domain, when compared to the native *Pf*GMPS structure (PDB-ID 4WIM), is rotated by 85° and translated by 3 Å, relative to the ATPPase domain (Figure 8). This rotated state is locked by a disulphide bond between Cys113 and Cys377, with the rotation leading to the lid loop reorganizing into the ATPPase active site. Unlike in other dimeric GMPSs, most of the residues of the lid loop, barring seven residues, are well-mapped in the structure of *Pf*GMPS_C89A/Gln [32]. In the structure of the double-mutant *Pf*GMPS_C89A_C113A (PDB-ID 7ZU9), the GATase domain is rotated by 170° and translated by 1.7 Å when compared to the native structure, and by 120° and 4 Å when compared to the structure of *Pf*GMPSC89A/Gln [33] (Figure 8). 

This extreme rotation of the GATase domain in the double mutant leads to an important loss of the secondary structure elements of the enzyme and to the collapse of the dimer into a monomer. Due to the mutation of Cys 113 to Ala, the mutant can no longer form a disulphide bridge; hence, there is continued spinning of the GATase unit, supporting domain rotation and its role in enzyme function [32,33]. Although to a lesser degree, GATase domain rotation has been observed in other GATs. In the crystal structure of *E. coli* glucosamine- 6-phosphate synthetase, bound to glucose-6-phosphate and modified by DON, a 23° GATase domain rotation is seen [60]. In addition, a small magnitude of a 3.6° rotation of the GATase domain has been seen in sulfate-bound CTP synthetase [61]. 

## 5. Substrate Contacts and Catalysis in the ATPPase Domain/Subunit

**Figure 9:**
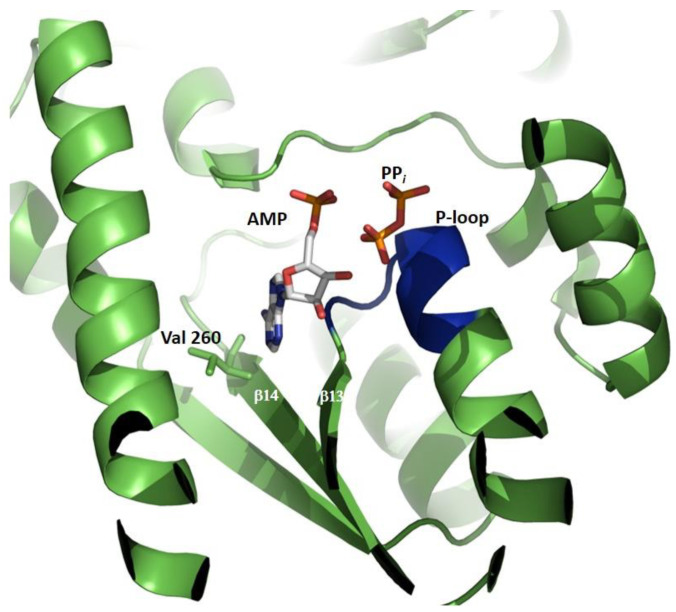
Close-up on AMP (white) and PP*_i_* (orange) binding to the ATPPase domain in the crystal structure of *Ec*GMPS (1GPM) in complex with AMP and PP*_i_*. The N6 amino group and N1 of the adenine ring of AMP hydrogen-bonds with backbone oxygen and nitrogen, respectively, of Val260. Close contacts with β-strands 13 (Lys229–Gly233) and 14 (Leu255–Asp261) exclude substitution at the C2 position and impart specificity solely for adenine. Interactions of PP*_i_* are with the P-loop (in blue) in the N-terminal ATPPase core sub-domain.

GMPSs belong to the family of N-type ATPPases, where the ATPPase domain/subunit binds the substrates ATP.Mg^2+^ and XMP and, *via* a phosphoryl transfer reaction, catalyzes the formation of the adenyl-XMP intermediate [46,61] that is aminated to GMP. An examination of the only available AMP*PP_i_-complexed *Ec*GMPS structure throws light on the tight specificity for adenine. The N6 amino group and N1 of the adenine ring of AMP are hydrogen-bonded with the backbone nitrogen and oxygen, respectively, of Val 260. Close contacts with β-strands 13 and 14 exclude substitution at the C2 position and impart a specificity solely for adenine. The interactions of PP*_i_* are with the P-loop in the N-terminal ATPPase core sub-domain [41] (Figure 9). 

### 5.1. C-Terminal Loop

**Figure 10:**
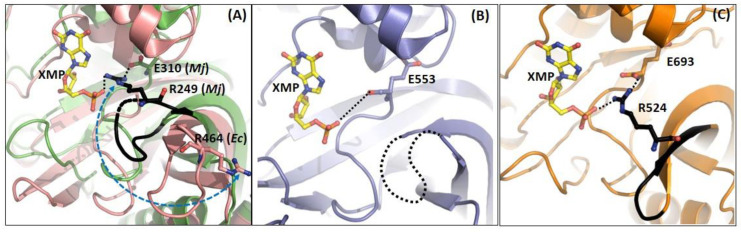
XMP binding to the ATPPase domain/subunit in the crystal structures of (**A**) *Ec*GMPS (1GPM, salmon) and *Mj*ATPPase (6JP9, green), (**B**) *Pf*GMPS (3UOW, blue), and (**C**) *Hs*GMPS (2VXO, orange). It should be noted that the *Ec*GMPS structure does not have bound XMP and the XMP seen in the figure is from the overlay of the structure of *Mj*ATPPase bound to the nucleotide. The C-terminal loop is displayed as a black coil.

In the structure of *Mj*ATPPase/XMP, O6 of xanthine contacts Arg102, and this is conserved in *Pf* and human GMPS_XMP (3UOW and 2VXO) structures, making this a key residue in discriminating XMP from other purine monophosphates. Arg102 is held in place by H-bonding to the α carboxy group of the last residue Glu310 of the C-terminal loop of the dimerization sub-domain [37]. The contacts of the C2 and C3 OH of the ribose unit of XMP are similar in both the *Pf*GMPS and *Mj*ATPPase structures [32,37]. The oxygens of the phosphate of XMP are hydrogen-bonded to the side-chain NH_2_ of Lys302 and backbone NH of Ile307 and Glu308. The side-chain COOH of Glu308 forms a salt bridge with Arg294 from the dimerization sub-domain of the neighboring chain and holds the C-terminal loop (298−310, *Mj*ATPPase numbering) in place for XMP binding [37]. Mutation of the residues Lys547, Glu553, and Arg539 in *Pf*GMPS, which correspond to Lys302, Glu308, and Arg294 in *Mj*ATPPase, to Leu result in a complete loss of Gln- and NH_4_Cl-dependent activity on account of impaired adenyl-XMP formation. In addition, GATase activation is reduced by >70%, suggesting that these mutants are defective in binding substrates [62]. Apart from this, there is a direct salt-bridge-like interaction between the phosphate of XMP and the side-chain of Arg249 from the neighboring chain. Arg249 and many residues in the loop (residues Gly243—Glu250) harboring this residue are invariant across two-subunit- and two-domain-type GMP synthetases. This loop shows conformational flexibility, being disordered in the structures of the GMPSs from *P. falciparum* (PDB ID: 3UOW), *Coxiella burnetii* (PDB ID: 3TQI), and *Thermus thermophilus* (PDB ID: 2YWB and 2YWC). However, in the structures of the native enzymes from *E. coli* and *Neisseria gonorrhoeae*, the Arg residue corresponding to the Arg249 of *Mj*ATPPase is positioned away from the active site of the neighboring protomer by more than 9 Å (Figure 10A), suggesting that the binding of XMP elicits a loop movement, enabling tight substrate binding. In addition, the Arg249 side-chain in *Mj*ATPPase also contacts the α carboxy of the last residue, Glu310, tightening the interactions of the C-terminal with the neighboring subunit and highlighting the importance of inter-subunit interactions in the dimers for enzyme function [37]. The presence of similar interactions of the C-terminal residues with XMP in the structure of *Pf*GMPS_XMP, along with the invariance of the residues involved [32], suggests a conserved mechanism of XMP binding (Figure 10B). In human GMPS, Arg524 in the inserted D1 sub-domain interacts with XMP bound to the same sub-domain (Figure 10C). 

### 5.2. Catalysis and Allostery: Role of Lid Loop Residues

In the structure of *Pf*GMPS_C89A/Gln, due to the rotation of the GATase domain, the N-terminal part of the lid loop occupies the ATPPase active site with a bending of the preceding helix 371–375, and blocks access to the site to which AMP, PP*_i_*, and XMP bind, as seen when superimposing the ligands as positioned in the structure of *Ec*GMPS (PDB ID 1GPM) onto that of *Pf*GMPS (PDB ID 4WIO) [32]. Indeed, this conformational change has implications in catalysis, as the residues Asp371 and Glu374 are positioned in the location expected for the adenyl-XMP bond. Studies on the mutants *Pf*GMPS_E374L and *Pf*GMPS_D317A have confirmed these structural observations. Activity measurements have indicated that, though *Pf*GMPS_D371A can bind the substrates and activate glutamine hydrolysis in the GATase domain, the adduct adenyl-XMP formation is almost completely compromised. In *Pf*GMPS_E374L, although the rate of adenyl-XMP formation is reduced only by a factor of 3.5, the mutant is completely devoid of glutamine-dependent GMPS activity, and the activation of GATase activity by the binding of substrates to the ATPPase domain is also fully abolished. The mutant, however, exhibits a 72-fold lower NH_4_Cl-mediated GMP formation. This indicates that Glu374 is involved in both adenyl-XMP formation and inter-domain cross-talk. In the structure of *Pf*GMPS, Glu374 contacts His20 in the GATase domain and the mutant His20Ala shows a 57% lower level of activation, suggesting inter-domain communication *via* the Glu374–His20 interaction [32]. 

Apart from the conserved Asp371 and Glu374, the lid loop has an invariant motif IK(T/S)HHN (residues 385–390 in *Pf*GMPS) [33] that contacts AMP when superposed from the *Ec*GMPS structure. While the mutants K386L, H388A, and H389A of *Pf*GMPS show greatly impaired NH_4_Cl- and Gln-dependent GMP formation, K386L and H389A are fully impaired in adenyl-XMP formation, and H388A shows a significantly lowered rate of adduct formation. The His mutants show a 50% lower GATase activation, but exhibit strong binding of both ATP and XMP, with *K*_d_ values in the low micromolar range. Concerning the Lys mutant, GATase activation is impaired to a much greater extent. These results establish that Lys386 is essential for substrate binding and that His388 and His389 play catalytic roles in the synthesis of the adenyl-XMP intermediate. These results clearly indicate that Lys386 participates in ATP binding, whereas His388 and His389 are involved in catalyzing the formation of the adenyl-XMP intermediate, where an increase in the electrophilicity at C2 due to adduct formation facilitates an attack by ammonia and the conversion of XMP into GMP [63]. 

While mutation of Thr387 to Ala impacts activity only marginally, mutation of Asn390 to Ala renders the enzyme completely inactive for GMP synthesis. The rate of adenyl-XMP formation is reduced, while the activation of the GATase domain is greatly enhanced. Taken together, these results indicate that Asn390 plays a key role in a step subsequent to adenyl-XMP formation involving the reaction of ammonia with the adduct to generate GMP. The distinct conformation of the lid loop in the structure of *Pf*GMPS_C89A/Gln throws light on all key steps in the catalytic process, *viz*., inter-domain communication, substrate affinity enhancement, the formation of the ammonia channel, and the expulsion of the reaction product [32,63]. Apart from contributing residues for catalysis, the lid loop, by covering the active site, shields the intermediate from attack by water and also provides a water-sealed pocket for ammonia tunneling.

### 5.3. Domain Rotation

As a consequence of domain rotation, as seen in the structure of *Pf*GMPS_C89A/Gln, Cys113, a residue in the GATase domain, comes proximal to and forms a disulfide bond with Cys377, a residue at the terminus of the helix holding Asp371 and Glu374. Mutation of Cys113 to Ala in *Pf*GMPS_C89A_C113A alters the values of the kinetic parameter *K*_m_ for NH_4_Cl, ATP, and XMP, supporting domain rotation and the placement of this region in the GATase domain in close proximity to a catalytically important segment in the ATPPase domain [32]. 

### 5.4. Domain Reorganization and Ammonia Tunneling

In all the available native and XMP- or AMP+PP*_i_*-bound structures of GMPSs from both prokaryotes and eukaryotes, the GATase active site is open to the solvent; hence, it does not provide a picture of the channel vital for the transport of ammonia. The 80° GATase domain-rotated structure of *Pf*GMPS_C89A/Gln is the first and only structure that provides a lead on the possible nature of the domain reorganization required to form the channel for the translocation of ammonia. Although a large magnitude domain rotation is observed, the GATase active site is not correctly positioned over the adenyl-XMP bond for a nucleophilic attack by ammonia. Information on the magnitude of the GATase rotation required to position the GATase active site over the adenyl-XMP bond comes from molecular dynamics (MD) simulation studies, wherein a 50° rotation of the GATase domain, starting from the native structure, leads to optimal orientation [32].

## 6. Determinants of Domain Cross-Talk and Allostery

### Residues on Interdomain Interface Helices α1, α11, and α12

**Figure 11:**
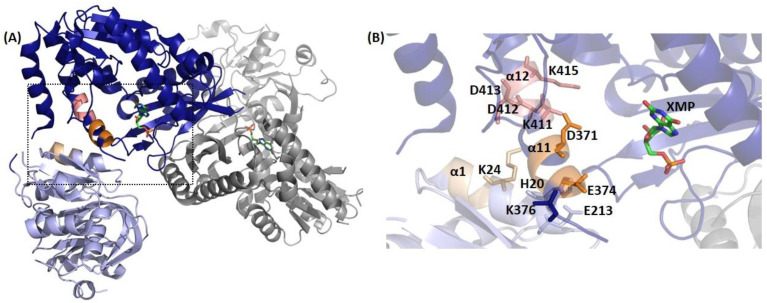
Interfaces important to cross-talk within GMPS, here exemplified by the crystal structure of *Pf*GMPS (3UOW), with (**A**) the GATase domain of one monomer colored in light blue and the ATPPase domain of the same monomer colored in dark blue. Motifs (K/R)(K/R)XRE (KRLNN in *Pf*), DXXES, and KD(D/E)V(K/R) are highlighted in wheat, orange, and salmon, respectively. The second monomer is colored in grey and XMP ligands are shown as sticks in green. (**B**) Close-up of boxed region in (**A**) with some of the residues having been observed as being essential in previous studies highlighted as sticks [32,33,63].

In the crystal structures of *Pf*GMPS and *Pf*GMPS/XMP, helix α1 from the GATase and helices α11 and α12 from the ATPPase largely comprise the interface between the two domains (Figure 11) and bear the motifs (K/R) (K/R)XRE (α1 motif), DXXES (α11 motif), and KD(D/E)V(K/R) (α12 motif) that are largely conserved, though in *Pf*GMPS, the sequence of the α1 motif is KRLNN. 

The conserved residues Asp371 of α11 and Lys411 and Lys415 of α12 are directed into the catalytic pocket in the ATPPase domain, where Asp371 and Lys411 contact the adenyl-XMP and PP*_i_*, respectively, whereas Lys415 is devoid of contacts with the ligands. While the role of Asp371 is associated with the catalysis of adduct formation, Lys411 is involved in substrate binding with the mutant *Pf*GMPS_K411L, showing a complete lack of adenyl-XMP and GMP formation, with a 70% drop in GATase activation. Similarly, with an increased *K*_m_ value for ATP and a 63% lower rate of adduct formation in *Pf*GMPS_K415L, Lys415 is also involved in substrate binding. Other residues on α11 and α12 of the ATPPase domain contact α1 of the GATase, with specific pairs being α1 Lys24 and α12 Asp412/Asp413, and α1 His20 and α11 Glu374. His 20 is not conserved and other inter-domain salt bridges are enzyme-specific. Mutations of Lys24, Asp412, and Asp413 all show an increase in the *K*_m_ value for the substrate glutamine, with the levels of GATase activation being also impaired, validating the structural contacts between α1 and α12 and their role in mediating domain cross-talk. In *Pf*GMPS, His 20 and Glu374 mediate the α1 interaction with α11, with Glu374 also playing an additional role in adenyl-XMP formation [32,63].

In the structure of *Pf*GMPS_C89A/Gln, helices α11 and α12 remain at the inter-domain interface, but due to the rotation of the GATase domain, contacts with α1 are lost. However, the Glu213/Lys376 salt bridge evident in the structure has been confirmed by mutagenesis. Mutation of Lys376 affects only the *K*_m_ for Gln, while mutation of Glu213 increases the *K*_m_ value for Gln and reduces GATase activation and the rate of adduct formation, suggesting a role for the 85° interface residues also in domain cross-talk [32,63]. 

As established in *Pf*GMPS, the binding of substrates to the ATPPase domain brings about a dramatic reduction in the *K*_m_ value for glutamine, leading to the activation of catalysis in the GATase domain [32]. The interacting residue pairs Lys24 (α1)-Asp412/413 (α12), Glu213 (GATase)-Lys376(α11), and His20 (α1)-Glu374 (α11) observed in the crystal structures have been experimentally confirmed to mediate GATase activation [63]. The involvement of helix α1 in mediating GATase/ATPPase cross-talk has also been observed in NMR experiments on the two-subunit GMPS from *M. jannaschii*, wherein residues in α1, along with the loop holding the catalytic triad, show changes in chemical shift upon binding to the substrate-bound ATPPase subunit [45]. Despite the distal location of Glu213 (close in sequence to catalytic His208 and Glu210) from the ATPPase active site in the *Pf*GMPS (3UOW) structure, the reduction in the rate of adduct formation by 41% supports domain rotation and relocation of Glu213 that, in turn, impacts catalysis in the ATPPase domain [32,63]. This is supported by studies on *Ec*GMPS, where a mutation of His 186 (corresponding to Glu213 in *Pf*GMPS) to Ala has led to a 50-fold increase in the *K*_m_ value for Gln and an uncoupling of the two reactions [30].

The large body of kinetic studies on structure-guided mutants has established that the interface helices α11 and α12 (and the lid loop connected to α11) have dual roles, *viz*., substrate binding and catalysis, and interdomain cross-talk, thus linking the catalysis in the ATPPase active site with GATase activation. The residues on α11 and α12 that mediate substrate binding are Lys386, Lys411, and Lys 415, while Asp371, His 388, and His 389 catalyze the formation of adenyl-XMP [32,63]. 

## 7. Structure of Two-Subunit *M. jannaschii* GMPS

### 7.1. Structure of the MjGATase–MjATPPase Complex

In two-subunit GATs, the GATase and acceptor/synthetase subunits form a tight complex, even in the absence of substrates, with *Mj*GMPS and *Ph*GMPS being exceptions. In the case of *Ph*GMPS, the complex of GATase and ATPPase subunits could be isolated in the presence of substrates, whereas the subunits of *Mj*GMPS associate transiently, even in the presence of ATP.Mg and XMP [37,45]. NMR studies have placed the interaction in the intermediate to fast exchange regimes of NMR timescales, with the steady-state *k*_cat_ value setting an upper limit of 0.5 sec for the time scale of subunit association, indicating that glutamine hydrolysis is very tightly regulated in *Mj*GMPS [37,45]. Despite the transient nature of subunit interaction, ammonia is channeled from the site of glutamine hydrolysis to the ATPPase active site [37]. Cross-linking of the two subunits in the presence of ATP.Mg and XMP, followed by in-gel trypsin digestion and mass spectrometry, along with cross-linking-guided docking, revealed that the structural elements and residues involved in inter-subunit interactions are conserved and similar to those in the 0° (native or XMP-bound) *Pf*GMPS and other dimeric GMPS structures [37]. The α1 (GATase) with α12 (ATPPase) interaction in all 0° rotated structures is conserved in *Mj*GMPS, with Lys 20 in helix G α1 of *Mj*GATase interacting with Asp167 of helix α6 of *Mj*ATPPase. In addition, the fusion of *Mj*GATase with *Mj*ATPPase as a single-chain protein yields an enzyme with characteristics similar to other single-chain dimeric GMPSs [37]. These studies indicate that the mechanisms of GATase activation, ammonia channeling, and GMP synthesis are similar in two-domain and in two-subunit GMP synthetases.

### 7.2. Stable Succinimide (SNN) in MjGATase

The GATase subunit of *Mj*GMPS has a highly stable succinimide arising from the deamidation of Asn109, the presence of which increases the structural stability of the protein at high temperatures [64]. The presence of SNN has the side-chain cyclized with the amide NH of the succeeding residue, leading to the torsion angle psi (Ψ) being restrained and generating a new route for hyperthermal stability. The SNN bearing *Mj*GATase remains folded at 100 °C and in 8 M guanidinium chloride, suggesting that sequences that stabilize succinimides from hydrolysis may be evolutionarily selected to confer extreme thermal stability [64]. SNNs, in general, are rapidly hydrolyzed to an Asp or β-Asp residue. However, in the structure of *Mj*GATase, the molecular basis of the stability of the SNN is revealed to be imparted by electrostatic shielding by the side-chain carboxylate group of its succeeding residue Asp110, as well as through the n/π* interactions between SNN109 and its preceding residue Glu108, both of which prevent water access to the SNN. The loop containing the SNN is locked in an α-turn that contributes to a reduction in protein flexibility. Several archaeal GATases have the SNN-forming tripeptide sequence (E(N/D)(E/D)) conserved, suggesting the role of this post-translational modification in the thermostability of the protein [55].

## 8. Conserved Catalytic Mechanism

**Figure 12:**
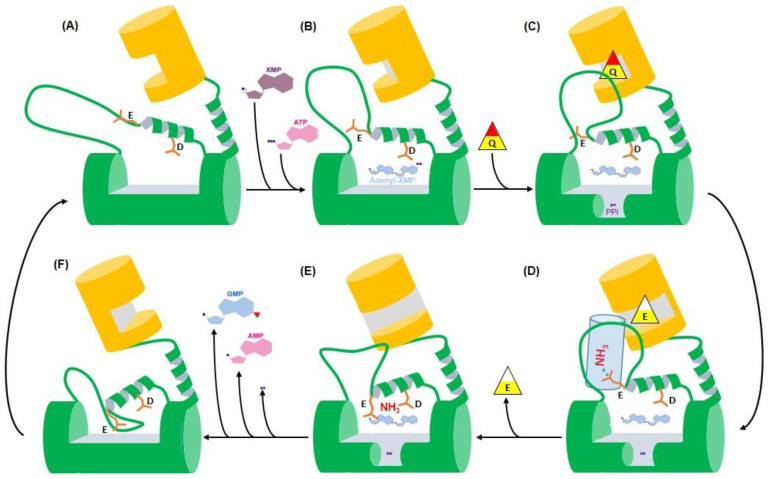
Scheme explaining the catalytic mechanism of GMPS (GATase domain in orange and ATPPase domain in green). (**A**) In the initial conformational state, the flexible lid loop is not present in the catalytic site of the ATPPase domain, and this allows binding of the substrates XMP and ATP. (**B**) Upon ATP and XMP binding, and concomitant with ATP hydrolysis, which results in the formation of AMP-XMP and PP*_i_*, the helix proceeding the flexible lid loop starts to bend, disrupts interactions between the GATase and ATPPase domains, reorients the flexible lid loop, and induces GATase domain rotation, creating a tighter binding site for the GATase substrate glutamine. (**C**) Glutamine binds (illustrated by a yellow and red “Q” triangle) and is deamidated, leading to a glutamate (illustrated by a yellow and white “E” triangle). (**D**) Due to channel formation (illustrated by a light blue cylinder), NH_3_ moves towards the ATPPase acceptor site driven by interaction with the ATPPase catalytic residues, Glu and Asp. (**E**) Attack of adenyl-XMP by NH_3_ to form GMP. (**F**) Subsequent to GMP formation, further GATase domain (orange) rotation to 80° from the native state results in the movement of the flexible lid loop into the active site, which induces the expulsion of the products, GMP, AMP, and PP*_i_*, and the return of the enzyme to its initial precatalytic state (**A**).

The availability of the various crystal structures of single-chain GMPSs in native and ligand-bound states, along with activity data on structure-guided, site-directed mutants, has enabled the formulation of the catalytic mechanism (Figure 12). Prior to the binding of ATP.Mg and XMP, the flexible lid loop is distal from the active site in the ATPPase domain, enabling substrate binding. The residues Asp371 and Glu374 (*Pf*GMPS numbering) on the helix preceding the lid loop, along with the residues on the invariant motif IK(T/S)HHN (residues 385–390 in *Pf*GMPS) on the lid loop, participate in ligand binding and catalysis of AMP-XMP formation [32,63]. This suggests that lid loop closure over the active site is concomitant with ligand binding. This movement of the lid loop leads to multiple alterations in the structure, *viz*., the bending of the preceding helix that harbors the residues Asp371 and Glu374, an alteration in GATase–ATPPase domain interaction, and the rotation of the GATase domain, with the latter two events leading to an enhanced affinity of Gln for the GATase active site and its subsequent hydrolysis, generating ammonia. In an intermediate conformation where the GATase domain has rotated by 50°, a channel that connects the GATase active site to the adenyl-XMP bond is formed, resulting in the movement of ammonia and an attack of the adenylated intermediate to form GMP [32]. The residues Asn390 and Glu374 (*Pf*GMPS numbering) play key roles in the step post adenyl-XMP formation [32,63]. Subsequent to GMP formation, a further domain rotation to 80° from the native state results in the movement of the lid loop into the active site, expulsion of the products, GMP, AMP, and PP*_i_*, and the enzyme returning to its precatalytic state. Taken together, the movement of the lid loop is the trigger that sets the enzyme in action. Studies on two-subunit GMPSs from *M. jannaschii* and *P. horikoshi* have indicated that the catalytic mechanism operating in this class is similar to that in the single-chain counterparts [32,37,63].

## 9. Conclusions

More than three decades of biochemical and structural studies have provided an in-depth understanding of the catalytic mechanism operating in GMPS. GMPS belongs to the G-type or class I triad family of amidotransferases [26], with the catalytic mechanism in the GATase pocket being similar to that in other triad GATs. However, the mechanism operating in the ATPPase pocket had remained elusive. The crystal structure of *Pf*GMPS_C89A/Gln exhibited a novel conformational state that enabled an in-depth structure–function analysis, leading to insights into this complex catalytic process [32]. Unlike other two-subunit GATs that are strongly associated in the native or substrate-bound states, the two-subunit GMPSs associate transiently, precluding the determination of the crystal structure of the functional enzyme complex. Cross-linking mass spectrometry on ligand bound *Mj*GMPS has shown that the subunits associate in a manner similar to domain interactions in single-chain GMPs [63]. Despite these milestones, certain questions remain unanswered. The availability of the structure of an inactive mutant of GMPS bound to ATP.Mg+XMP+Gln or the wild-type enzyme bound to a non-hydrolysable analog of adenyl-XMP will approach a complete understanding of the function of this glutamine amidotransferase. These structures may also enable the visualization of the ammonia tunnel. IMPDH, the enzyme preceding GMPS in the biosynthesis of GMP, is a target for immunosuppression with the inhibitor mycophenolic acid in clinical use. Similarly, GMPS could be exploited for the development of anticancer and antiprotozoal drugs. A key difference between the human and parasite GMPS is that the former is a monomer, while the latter a dimer with XMP binding involving residues across the subunits. Small molecules that impair protein association could serve as inhibitors of single-chain dimeric GMPS. Finally, the presence of a stable succinimide (SNN) in *Mj*GATase, contributing to hyperthermostability and the conservation of the SNN-bearing sequences in thermostable archaeal GATases, suggests a widespread occurrence and merits investigation.

## Figures and Tables

**Table 1:** Kinetic parameters for GMPSs from different organisms.

Organism [ref]	*k*_cat_ (s^−1^)	*K*_m_				
		ATP (μM)	XMP (μM) (Hill Coefficient n)	Gln (μM)	NH_4_^+^ (mM)	Mg^2+^
*E. coli* [44]	---	104 ± 44	166 ± 43	---	103	---
*E. coli* [30] (Gln as NH_3_ source)	23 ± 1 (XMP) 13.7 ± 0.5 (Gln)		53 ± 7	1.6 ± 0.3 mM		
*E. coli* [30] (NH_4_Cl)	13.3 ± 0.3		43 ± 4			
*H. sapiens* [34]	5.4	132 ± 7	K_0.5_ 35.6 ± 1.8 (n = 1.48)	406 ± 49 μM	174 ± 21 mM	K_0.5_ 1780 ± 70 μM n = 4.14 ± 0.77
*T. brucei* [5]	4.7	200	8.8	240 μM		
*M tuberculosis* [40]	2.3	27 ± 2	K_0.5_ 45 ± 1 (n = 2.4)	1.24 mM	K_0.5_ 26 mM	K_0.5_ 1.18 ± 0.03 mM n = 2.67
*P. falciparum* [28,38]	0.43 (Gln)	260 ± 38	16.8 ± 2	472 ± 69 μM	10.8 mM	K_0.5_ 2090 ± 30 μM n = 4.4
*M. jannaschii* (70 °C)	1.94 ± 0.02 (Gln)	452 ± 3	61 ± 3	520 ± 8 μM		K_0.5_ 1.84 ± 0.01 mM n = 2.05 ± 0.07
*MjATPPase* (70 °C) [37]	1.91 ± 0.02 (NH_4_^+^)	447 ± 5	30 ± 2	NA	4.1 ± 0.2 mM	K_0.5_ 1.75 ± 0.02 mM n = 2.45 ± 0.05
*C. neoformans* [7]	0.4	77.5 ± 6.0	65.9 ± 13.0	1130 ± 162 μM		K_0.5_ 1289.0 ± 66.0 μM n = 2.2 ± 0.2
*A. fumigatus* [43]	1.6 ± 0.1	245 ± 15	ND	2693 ± 119 μM		*K*_m_ = 1230 ± 140 μM

**Table 2:** Existing three-dimensional structures of GMP synthetases, and isolated domains/subunits hereof. The PDB-IDs related to the relative studies are given in bold.

Organism	Native/Mutant	Ligand Bound	GATase Domain	GATase Ligand Bound
**Single chain**				
*A. baumannii* AB5075-UW		Mg^2+^, PO_4_^3−^, Cl^−^; **7SBC**		
*A. fumigatus* Af293	**7MO6**			
*C. burnetii*	**3TQI**			
*E. coli*		AMP, citrate, Mg^2+^, PO_4_^3−^, PP*_i_*; **1GPM**		
*H. sapiens*		XMP; **2VXO**	**2VPI**	
*N. gonorrhoeae*		Mg^2+^; **5TW7**		
*P. falciparum*	**4WIM** C89A_C113A; **7ZU9**	C89A with Gln; **4WIO** XMP & Ca^2+^; **3UOW**	NO_3_^−^; **4WIN**	
*T. thermophilus*	**2YWB**	XMP; **2YWC**		
**Double chain**				
*M. jannaschii*	**2LXN**	XMP; **6JP9**	**7D40** D110G; **7D96** and N109P; **7D97**	Acivicin; **7D95**
***P. horikoshii*** **OT3**	**3A4I/2DPL**		**2D7J/1WL8**	

## Data Availability

Not applicable.

## Supplementary Materials

The following supporting information can be downloaded at: https://www.mdpi.com/article/10.3390/biom13091379/s1, Figure S1: Sequence alignment of representatives of GMPSs. The sequences of *MjGA*Tase and *Mj*ATPPase subunits have been fused in the alignment. The sequence alignment has been performed using the program ESPript [65]; Figure S2: Subdomains D1 (blue) and D2 (green) in *Hs*GMPS display very similar fold with a rmsd of 2.2 Å.; Figure S3: Topology diagram of the *Mj*ATPPase subunit structure. The structure of the dimerization subdomain and the inter-subunit contacts in the dimer of the *Mj*ATPPase subunit is similar to the counterparts in the two domain GMPS structures.
