# Supplementary material for: GMP Synthetase: Allostery, Structure, and Function
Source: Biomolecules. 2023 Sep 12;13(9):1379. doi: 10.3390/biom13091379 (PMC10526850; doi:10.3390/biom13091379)
Supplement: Supplementary file 1 [file biomolecules-13-01379-s001.zip › biomolecules-2533156-supplementary.pdf]

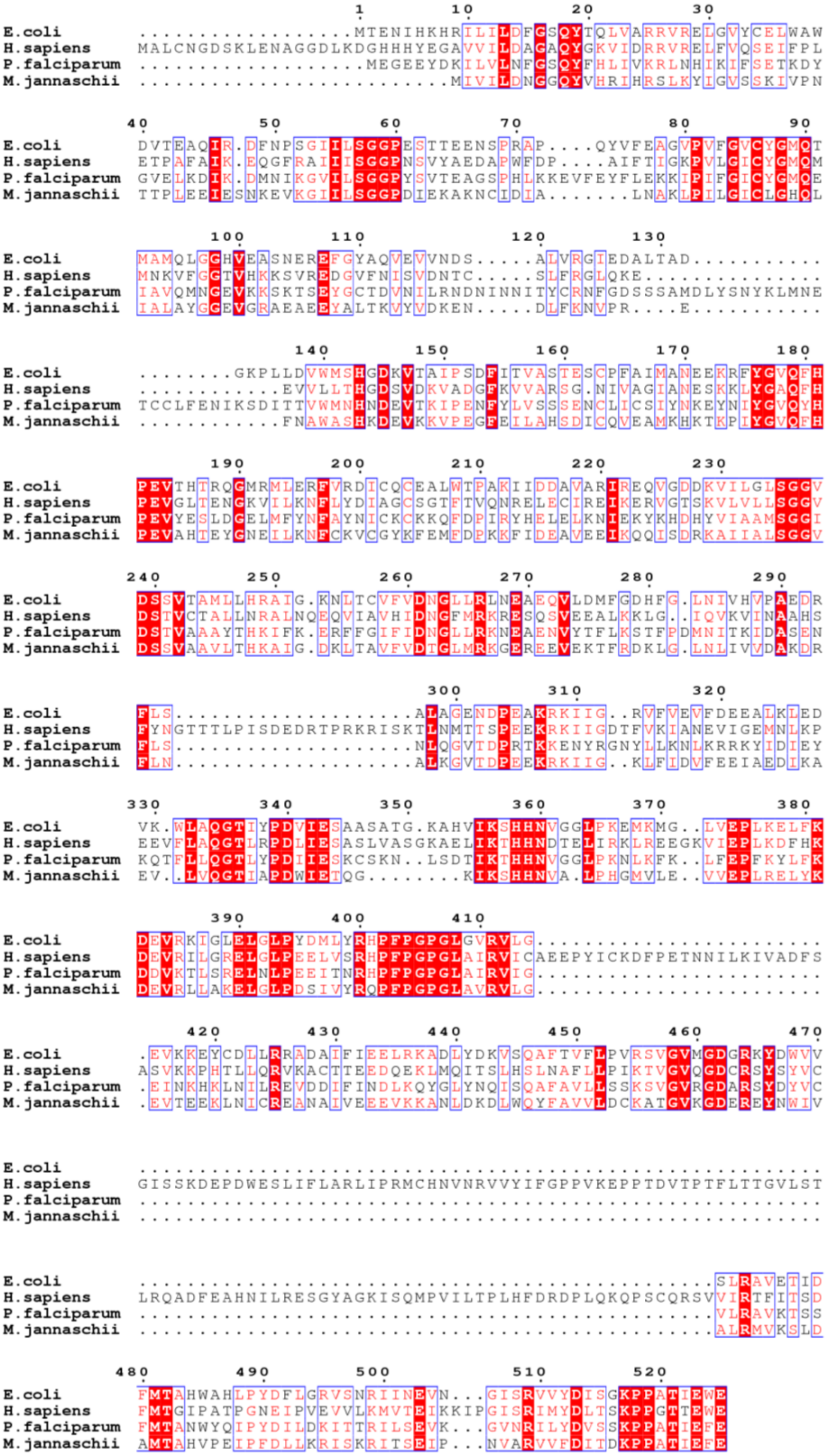

Supplementary Figure S1. Sequence alignment of representatives of GMPSs. The sequences of *Mj*GATase and *Mj*ATPPase subunits have been fused in the alignment. The sequence alignment has been performed using the program ESPript [65].

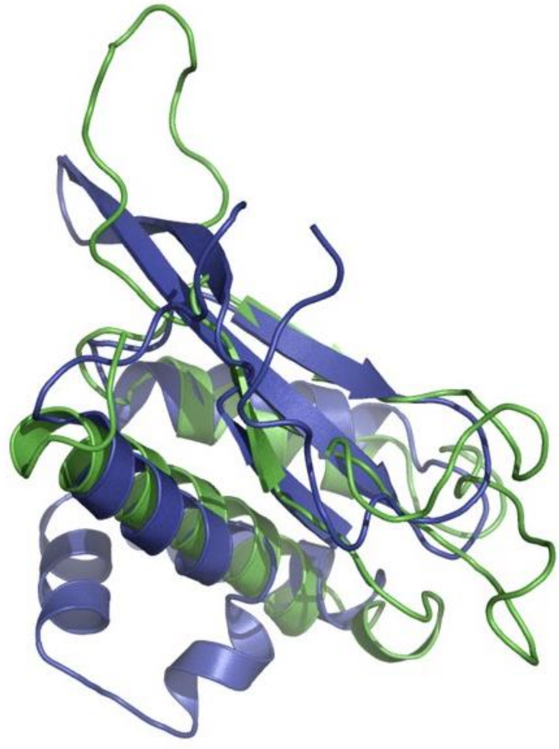

Supplementary Figure S2. Subdomains D1 (blue) and D2 (green) in *Hs*GMPS display very similar fold with a rmsd of 2.2 Å.

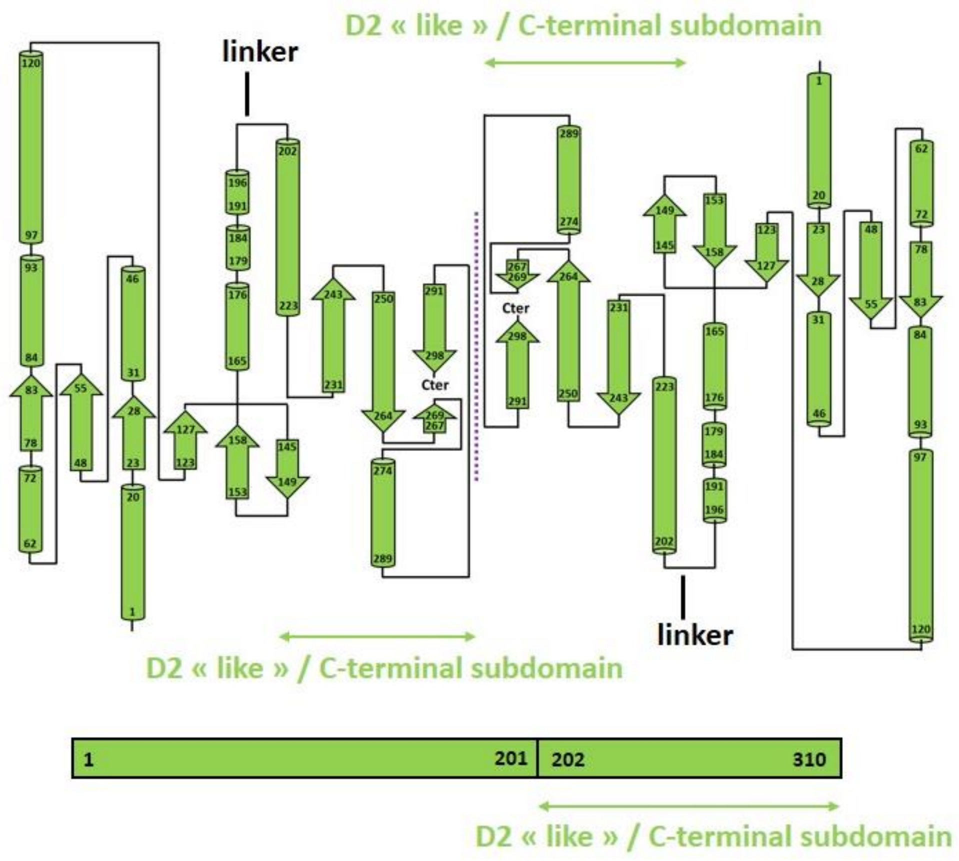

Supplementary Figure S3. Topology diagram of the *Mj*ATPPase subunit structure. The structure of the dimerization subdomain and the inter-subunit contacts in the dimer of the *Mj*ATPPase subunit is similar to the counterparts in the two domain GMPS structures.
